# Effect of potassium-rich maize (*Zea mays* L.) straw extract on salt stress mitigation in wheat (*Triticum aestivum* L.) seedlings

**DOI:** 10.3389/fpls.2026.1796229

**Published:** 2026-04-30

**Authors:** Changhai Shi, Aiguo Wang, Xinghua Geng, Lingyan Li, Xuejie Wan, Nicola Cannon, David O’Connor, Xianmin Chang, Yiguo Liu

**Affiliations:** 1 College of Agronomy, Qingdao Agricultural University, Qingdao, Shandong, China; 2 Royal Agricultural University, Cirencester, United Kingdom

**Keywords:** allelopathy, maize straw extract, root characteristics, salt stress, stress physiology

## Abstract

Saline soil significantly hinders crop growth potential, reducing grain yields. Applying maize (*Zea mays* L.) straw extract, rich in potassium ions (K^+^), in saline soil regions may help alleviate salt stress in wheat (*Triticum aestivum* L.) seedlings by rendering higher K^+^ levels and reducing magnesium ion (Mg^2+^) exudation. This study examines the effects of maize straw extract on the growth and physiology of wheat seedlings (Qingmai 6) under salt stress, applying low (0.01 g mL^-1^) and high (0.05 g mL^-1^) doses of maize straw extract under salinity conditions of low (1.0%) and high (1.5%) salt (NaCl) solutions. We found that seedlings treated with high-concentration maize straw extract under low salt conditions exhibited significantly greater total root length than without straw extract. The straw extract also helped maintain a favourable ion balance and improved the K^+^/Na^+^ and Mg^2+^/Na^+^ ratios in the wheat seedlings. Furthermore, under high salt stress conditions, the application of straw extract resulted in a notably lower proline content, indicating a significant alleviating effect. These findings highlight the importance of conducting additional research to explore the effectiveness of straw extract as a potential solution for salt stress in wheat seedlings.

## 1 Introduction

Saline soil disrupts ion balance. It also affects reactive oxygen species homeostasis and osmotic adjustment. Consequently, it hinders crop growth potential and reduces grain yields (Bhat et al., 2024; Singh et al., 2021). Approximately 8.7% of the Earth’s surface area is impacted by soil salinization, with China’s saline soil covering 25% of its arable land area, amounting to 36 million ha (Vargas, 2022; Mao et al., 2016). It is, therefore, imperative to research practical management approaches for saline arable lands.

Research has shown that returning straw residues can improve saline soils’ physical and chemical properties. Chen et al. (2020), for instance, showed that returning granularized maize straw improved the soil organic matter (OM) content and water-holding capacity of saline-alkali soil while reducing the soil pH, electrical conductivity and bulk density. This improvement occurred during the decomposition of the straw of the previous crop and sensitized substances that affect the soil microorganisms and subsequent crop growth (Aslam et al., 2017; Han et al., 2017). Meanwhile, straw’s influence on subsequent crop growth is affected by many factors. The extracts of wheat straw stimulated the germination of *Orobanche minor* (Sm.) seeds, with the growth stage of the wheat material used in the extract, the extraction solvent type, and the extract’s concentration had different effects on seed germination of *O. minor* (Dong et al., 2012).

The effects of the extracts vary between crops and cellular mechanisms of the same crop. Zhang et al. (2024) reported that the allelopathic effects of the rice (*Oryza sativa* L.) straw amendment on Chinese cabbage (*Brassica chinensis* L.) were greater than those of the maize straw and wheat straw amendments. Water extracts of *Pugionium maizeutum* (L.) leaves, peel, and branches inhibited the growth of Chinese cabbage seedlings, whilst the aqueous leaf extracts had a higher inhibition degree than peel and branch extracts (Bao et al., 2015). The allelopathic strength of water extracts of *Prangos ferulacea* L. stems on *Trifolium resupinatum* (L.) was the highest, followed by the extracts of leaves and flowers (Sadeghi and Bazdar, 2018). Moreover, the allelopathic effect of straw extract was concentration-dependent (Dong et al., 2024). The lowest concentration (10 to 100 ppm) of redroot pigweed (*Amaranthus retroflexus* L.) extract promoted the growth of wheat and kidney beans (*Phaseulus vulgaris* L.). Meanwhile, the extract at a high concentration (1000 ppm) promoted wheat growth but inhibited kidney beans (Aslam et al., 2017; Hamideh et al., 2015). redroot pigweed extract at the same concentration demonstrated differences in allelopathic effects on different crops. Studies have also shown that different crops respond differently to straw extracts (Katarzyna et al., 2018). When winter wheat is grown on saline-alkali land, germination and seedling development stages are important as they influence the crop canopy architecture and yield (Dong et al., 2019; Zaman et al., 2025; Guo et al., 2026). When maize straw is returned to the field, it influences the root-growing environment of winter wheat during the seedling stage.

Studies have shown that the application of low concentrations of sorghum (*Sorghum vulgare* Pers.) and sunflower (*Helianthus annuus* L.) straw aqueous extracts delayed ageing and improved yield in wheat (Bajwa et al., 2020). The allelopathic momilactones A and B, which are abundant in rice straw, have been confirmed to alleviate salt stress in rice (Xuan et al., 2016). However, to date, no study has directly investigated the effects of maize straw extract on wheat growth under salt stress. Although Zhang et al. (2024) examined maize straw in the context of clubroot disease control in Chinese cabbage, their study did not involve salt stress or wheat seedlings, leaving a clear research gap. In saline-alkali lands in the Yellow River Delta, wheat fields are irrigated with large volumes of Yellow River water to flush the salt and protect seedlings. However, the allelopathic effect of maize straw extract on wheat growth in saline-alkali land is unclear under double cropping of wheat and maize in a year. Therefore, we propose the hypothesis that maize straw extract, rich in K^+^, alleviates salt stress in wheat seedlings by improving ion balance (increasing K^+^/Na^+^ ratio, reducing Mg²^+^ exudation) and osmotic regulation (reducing proline content). Therefore, a water culture experiment was carried out to investigate the impact of maize straw extract on wheat morphology and physiological characteristics under salt stress at the seedling stage. This study is the first to systematically examine the effects of maize straw extract on wheat seedlings under varying salt stress intensities, aiming to provide a theoretical basis and practical reference for wheat cultivation in saline-alkali soils.

## 2 Materials and methods

### 2.1 Straw extract

Crushed maize straw (variety Zhengdan 958) was obtained from the Jiaozhou Modern Agricultural Science and Technology Demonstration Park of Qingdao Agricultural University in Shandong, China. The maize straw extract was prepared by steeping 100 g of straw in 1 L of distilled water at 4 °C for 48 hours. The resulting extract was filtered, diluted to 0.01 g mL^-1^ and 0.05 g mL^-1^ concentrations, and stored at 4 °C.

Previous studies have shown that organic compounds in straw leachate are important allelochemicals (Aslam et al., 2017; Xuan et al., 2016). To this end, gas chromatography-mass spectrometry was used to quantify semi-volatile organic compounds in the straw extract (**Table 1**). For this, 250 mL of straw extract was mixed with 30 mL of methylene chloride and hexane (1:1 ratio) and vortexed for 10 minutes, left to settle, and the organic phase removed. The process was repeated, and the organic phases were mixed together and condensed (to < 5 mL) by swirl distillation before being purified by Florisil-phase extraction and concentrated to 1.0 mL for analysis by GC/MS (Agilent 7890B + 5977B, Agilent Technologies, USA). The organic compounds in the straw extract were identified by searching the NIST2014 mass spectral library, comparing the standard spectra in the mass spectrometry database, and analyzing the artificial spectra combined with literature. The compounds were identified based on structure similarity of >80% with **Table 1** of Chen et al. (2019).

**Table 1:** Semi-volatile organic compounds (SVOCs) in the maize straw extract identified by GC/MS.

Retention time (min)	Compound name	Molecular formula	Molecular weight (g mol^-1^)	Similarity index
8.11	n-Decane	C10H22	142	850
9.04	Phenylcarbinol	C7H8O	108	900
10.27	Guaiacol	C7H8O2	124	946
10.8	Phenethyl alcohol	C8H10O	122	927
11.99	Benzoic acid ethyl ester	C9H10O2	150	899
12.54	Dodcane	C12H26	170	842
14.26	Indole	C8H7N	117	871
14.85	Ethyl dodecanoate	C14H28O3	244	661
15.67	Ethyl cinnamate	C11H12O2	176	921
15.77	Methyl cinnamate	C10H10O2	162	924
16.99	1,2-Benzenedicarboxylic acid dimethyl ester	C10H10O4	194	919
17.15	Ethyl 3-phenyl propenoate	C11H12O2	176	921
18.63	2-Propenoic acid,3-phenyl-1-methylethyl ester	C12H14O2	190	838
18.78	3,5,5-Trimethyl-4-butenylidene-2cyclohexen-1-one	C13H18O	190	828
19.04	3-Methyl-2(3H)-benzothiazolethione	C8H7NS2	181	689
24.13	6,10,14-Trimethylpentadecane-2-one	C18H36O	268	808
30.31	Ethyl oleate	C20H38O2	310	837
31.19	N-Pheny-2-naphthylamine	C16H13N	219	869
35.73	n-Tetracontane	C44H90	618	752

Chromatographic separation was undertaken using a DB-5ms (30 m × 0.25 mm, 0.25 m) column. The temperature program was set at 40 °C for 1 min, then increasing at 30 °C min^-1^ to 130 °C, 10 °C min^-1^ to 280 °C, and 20 °C min^-1^ to 310 °C, which was maintained for 15 min. The carrier gas was helium at a flow rate of 0.95 mL min^-1^ (no split mode). Mass spectrometry used an ion source temperature of 230 °C, electron energy of 70 eV, four-stage rod temperature of 150°C, quality scanning range (*m*/z) of 40–500, and a solvent removal time of 4.7 min.

### 2.2 Cultivation

Wheat seeds of uniform size were soaked in 1% sodium hypochlorite (NaOCl) solution for 15 min and rinsed with sterile distilled water. This experiment used a two-factor completely randomized factorial design, with salt stress level and straw extract concentration as the two factors. Salt stress was applied at three levels (none, low, and high), and straw extract at three concentrations (0, 0.01, and 0.05 g mL^-1^), resulting in nine treatment combinations (**Table 2**). One hundred seeds were placed in petri dishes (plates) with two layers of filter paper with 3 mL of straw extract (0.01 g mL^-1^ or 0.05 g mL^-1^) and 3 mL of NaCl solution (171.1 mM or 256.7 mM, equivalent to 1.0% and 1.5% NaCl, respectively, with an electrical conductivity of approximately 15.57 and 22.97 mS cm^-1^) added to the plates (**Table 2**). These concentrations were selected based on previous studies on salt stress in wheat (Li and Gao, 2018; Wang et al., 2025) The final NaCl concentrations in the medium were 0, 85.6, and 128.3 mM for the none, low, and high salt levels, respectively. Distilled water was applied without salt or straw extract for the control sample. Each treatment was undertaken in triplicate.

**Table 2:** Experiment treatments.

Salt level	Treatment	Salt solution (SA) mM NaCl	Straw extract (ST) g mL^-1^	[NaCl] (medium) mM
None	CK	0%	0	0%
	STL	0%	0.01	0%
	STH	0%	0.05	0%
Low	SAL	171.1	0	85.6
	SAL-STL	171.1	0.01	85.6
	SAL-STH	171.1	0.05	85.6
High	SAH	256.7	0	128.3
	SAH-STL	256.7	0.01	128.3
	SAH-STH	256.7	0.05	128.3

Distilled water was used to replace the 0 mM salt concentration and 0 mL straw extract in equivalent volumes.

The plates were placed in an RDN-400D-P artificial climate chamber under dark conditions for 24 h at 25 °C and 70% relative humidity for 3 days and then kept under 4,000 lx illumination from day 4. The dishes were weighed daily with distilled water added, when necessary, to ensure a constant solution concentration. On day 4, the number of germinated seedlings was recorded to calculate the germination potential. The germination rate was calculated on day 7. Seedlings were collected to determine their morphological and physiological indexes on day 10.

### 2.3 Seedling analysis

On day 4, the germination potential was calculated as the percentage of total seeds that had germinated (Germination potential (%) = (Number of seeds germinated by day 4/Total number of seeds tested) × 100). On day 7, the germination rate was calculated as the percentage of normally germinated seeds (Germination rate (%) = (Number of normally germinated seeds by day 7/Total number of seeds tested) × 100).

On day 10, the coleoptile height of 20 seedlings from each dish was measured. Afterward, the shoot and root tissues were separated, oven-dried at 105°C for 30 min, followed by drying at 80°C until a constant weight was achieved. Fresh wheat seedling leaves (1 g) were mixed with phosphoric Buffered Saline (PBS) and quartz sand and ground in an ice bath. The homogenate was centrifuged at 12,000 g for 10 min at 4 °C, and the supernatant made up to 10 mL with additional PBS.

#### 2.3.1 Antioxidant enzyme assays

Superoxide dismutase (SOD) activity was determined using the nitroblue tetrazolium (NBT) photoreduction method following Gao and Cai (2019). Briefly, 0.2 mL of enzyme extract was added to a reaction mixture (total volume 6 mL) containing 3 mL of 50 mmol L^-1^ phosphate buffer, 0.6 mL of 13.37 mmol L^-1^ methionine, 0.6 mL of 75 μmol L^-1^ NBT, 0.6 mL of 0.1 mmol L^-1^ EDTA, 0.6 mL of 2.5 μmol L^-1^ riboflavin, and 0.4 mL of distilled water. The mixture was illuminated at 25°C under 4,000 lx for 20 min. Absorbance was measured at 560 nm, with a blank kept in the dark as a reference. One unit of SOD activity (U) was defined as the amount of enzyme required to inhibit 50% of NBT photoreduction. SOD activity was calculated as: *SOD activity (U g^-1^ FW) = (A-A*_s_*) × V*_t_*/(A*_o_*×* 0.5 *× W × V*_s_*)*, where A_o_ is the absorbance of the control, A_s_ is the absorbance of the sample, V_t_ is the total volume of the extract, V_s_ is the volume of extract used for the assay, and W is the fresh weight of the sample.

Peroxidase (POD) activity was assayed using the guaiacol method following Gao and Cai (2019). The reaction mixture (3 mL) consisted of 100 mmol/L phosphate buffer (pH 6.0) containing 28 μL of guaiacol and 19 μL of 30% H_2_O_2_. The reaction was initiated by adding 1 mL of enzyme extract, and the increase in absorbance at 470 nm was recorded immediately. A control cuvette contained 1 mL of phosphate buffer instead of the enzyme extract. One unit of POD activity (U) was defined as a 0.01 increase in absorbance per minute. POD activity was calculated as: *POD activity (U g^-1^ FW min^-1^) =* Δ*A*_470_*× V_t_/(*0.01 *× W × V*_s_*× t)*, where Δ*A*_470_ is the change in absorbance over the reaction time t (min), V_t_ is the total volume of the extract, V_s_ is the volume of enzyme extract used for the assay, and W is the fresh weight of the sample.

#### 2.3.2 Osmotic regulator measurements

Proline content was determined using the ninhydrin colorimetric method following Gao and Cai (2019). Fresh wheat seedling leaves (0.5 g) were ground with 3% sulfosalicylic acid, and the homogenate was boiled in a water bath for 10 min. After cooling and centrifugation, 2 mL of the supernatant was mixed with 2 mL of glacial acetic acid and 2 mL of ninhydrin reagent. The mixture was boiled for 30 min, then cooled rapidly. The red product was extracted with toluene, and the absorbance of the toluene phase was measured at 520 nm. Proline concentration was calculated using a standard curve [(*y = 0.1599x-0.0129*, R^2^ = 0.9871, where y is absorbance and x is proline content in (μg)]. Proline content was then calculated as: *Proline content(μg g^-1^ FW) = C × V*_t_*/(W × V*_s_*)*, where C is the proline amount (μg) derived from the standard curve, V*_t_* is the total volume of the extract, V*_s_* is the volume of extract used in the reaction, and W is the fresh weight of the sample.

Soluble sugar content was determined using the anthrone colorimetric method following Gao and Cai (2019). Fresh wheat seedling leaves (0.2 g) were extracted twice with 80% ethanol in an 80°C water bath. The supernatants were combined and adjusted to a final volume of 10 mL. A 0.2 mL aliquot of the extract was mixed with 2 mL of 0.2% anthrone in concentrated sulfuric acid and heated in a boiling water bath for 10 min. After cooling, absorbance was measured at 620 nm. Soluble sugar concentration was calculated using a standard curve [(*y = 0.0006x + 0.0846*, R^2^ = 0.8938, where y is absorbance and x is sugar content in (μg)]. Soluble sugar content was then calculated as: *Soluble sugar content(mg g^-1^ FW) = C × V_t_/(W × V_s_ ×* 1000*)*, where C is the sugar amount (μg) derived from the standard curve, V_t_ is the total volume of the extract, V_s_ is the volume of extract used for the assay, and W is the fresh weight of the sample.

#### 2.3.3 Lipid peroxidation assay

Malondialdehyde (MDA) content was determined using the thiobarbituric acid (TBA) method following Gao and Cai (2019). Fresh wheat seedling leaves (0.2 g) were ground with 5 mL of 10% trichloroacetic acid (TCA). After centrifugation, 2 mL of the supernatant was mixed with 2 mL of 0.6% TBA and heated in a boiling water bath for 15 min. The mixture was then cooled on ice, centrifuged, and the absorbance was measured at 450, 532, and 600 nm. MDA content was calculated as: *MDA content(μmol g^-1^ FW) = [*6.45 *× (A*_532_*-A*_600_*) –* 0.56 *× A*_450_*] × V_t_/(W × V*_s_*)*, where V_t_ is the total volume of the extract, V_s_ is the volume of extract used for the reaction, and W is the fresh weight of the sample.

#### 2.3.4 Root activity

An additional 20 seedlings were collected from each dish, and the root systems were scanned using EPSON SCAN and analysed using WinRHIZO Pro 2012b to analyze their root morphological index. Root activity was measured using the 2,3,5-triphenyltetrazolium chloride (TTC) reduction method. Fresh roots (0.3 g) were immersed in a mixture of 0.4% TTC and phosphate buffer (pH 7.0) and incubated in the dark at 37°C for 2 h. The reaction was terminated by adding 1 mol L^-1^ sulfuric acid. The roots were then dried with filter paper, and triphenylformazan (TTF) was extracted by grinding with ethyl acetate. The extract was adjusted to a final volume, and its absorbance was measured at 485 nm. TTF content was calculated using a standard curve [*y = 0.0812x-0.0456*, R^2^ = 0.9802, where y is absorbance and x is TTF content (μg)]. Root activity was calculated as: *Root activity(μg TTF g^-1^ FW h^-1^) = C/(W × t)*, where C is the TTF amount (μg) derived from the standard curve, W is the fresh weight of the roots (g), and t is the reaction time (h, Gao and Cai, 2019).

#### 2.3.5 Ion content analysis

Ground dry leaf (0.5 g) was digested in a strong acid solution of 10 mL concentrated nitric acid and 2 mL perchloric acid. The concentrations of sodium (Na), potassium (K), calcium (Ca), and magnesium (Mg) were determined using an inductively coupled plasma-optical emission spectrometer (ICP-OES, PerkinElmer, Waltham, MA, USA). The working conditions were: RF power 1300 W, cooling gas flow 12 L min^-1^, auxiliary gas flow 0.2 L min^-1^, nebulizer gas flow 0.8 L min^-1^, and integration time ≥5 s. The characteristic spectral lines were Na 589.592 nm, K 766.491 nm, Ca 317.933 nm, and Mg 285.213 nm. Standard curves were prepared using mixed standard solutions of 0.05, 0.1, 0.5, 1.0, and 5.0 mg/L. The standard curves were: Na: *y = 33124.12x + 5926.85* (R^2^ = 0.9992), *K: y = 3544.41x + 3424.22* (R^2^ = 0.9994), Ca: *y = 21168.64x + 495.58* (R^2^ = 0.9998), Mg: *y = 50650.01x + 276.84* (R^2^ = 0.9994), where y is the emission intensity and x is the element concentration (mg L^-1^). The element content in the samples was calculated as: *Element content(mg g^-1^) = C × V/(W ×* 1000*)*, where C is the element concentration (mg L^-1^) derived from the standard curve, V is the final volume (mL), and W is the dry weight of the sample (g, Hong et al., 2023).

### 2.4 Statistics

SPSS V 18.0 (IBM Inc., USA) was used to undertake the significant tests, and Duncan’s new multiple range test was used for multiple comparisons. The difference is considered significant at the *P* < 0.05 level.

## 3 Results

### 3.1 Germination

**Table 3** shows that all treatments without salt stress exhibited high germination potential (97-98%) with no significant differences. Under low salt stress, the SAL-STL treatment maintained a high % germination potential of 97% and a germination rate of 95%. The SAL-STH treatment showed a high germination potential of 95%. However, the germination rate decreased to 90%, which was significantly lower than SAL-STL but not significantly different from SAL. Under high salt stress conditions, all treatments had notable decreases in germination potential compared to CK (to 94%) and germination rates (to 90-91%), with no significant difference rendered by the straw extracts. This indicates that the inhibitory effect of straw extract on germination is concentration-dependent. A high concentration may interfere with radicle emergence.

**Table 3:** Effect of straw extract on germination under various salt stress conditions.

Salt level	Treatment	Germination potential	Germination rate
None	CK	98%^a^	97%^a^
	STL	97%^a^	96%^a^
	STH	98%^a^	94%^abc^
Low	SAL	95%^bc^	94%^abc^
	SAL-STL	97%^ab^	95%^ab^
	SAL-STH	95%^bc^	90%^c^
High	SAH	94%^c^	91%^bc^
	SAH-STL	94%^c^	90%^c^
	SAH-STH	94%^c^	91%^bc^

Different lowercase letters indicate significant differences (*p* < 0.05).

### 3.2 Plant height and biomass

**Table 4** shows CK exhibited the tallest average plant height (3.24 cm plant^-1^), significantly greater than all other treatments, including those with straw extract but without salt stress. The CK also had the largest average shoot mass (0.208 g plant^-1^ DW), but there were no significant differences with STL or STH. Root mass was greater (but not significantly; *p* > 0.05) in the STL and STH treatments (0.161-0.162 g plant^-1^ DW) compared to the CK (0.151 g plant^-1^ DW), resulting in higher root-to-shoot mass ratios compared to the CK. Under low salt stress conditions, plant height significantly decreased across all treatments (2.18-2.35 cm) compared to CK, with SAL-STL and SAL-STH treatments showing no significant improvement over SAL. The straw extract treatments also had no significant difference on shoot and root biomass compared to SAL. Under high salt stress, plant height was significantly reduced across all treatments compared to CK, with SAH-STL and SAH-STH showing the smallest heights (1.64-1.78 cm), significantly shorter than the plant height in SAH without straw extract applied (2.14 cm). Shoot and root masses were lower in the SAH treatment compared to SAH-STL and SAH-STH, although the differences were not significant. Plant height decreased while the root-to-shoot ratio increased. This reflects that seedlings prioritize resource allocation to roots under stress.

**Table 4:** Effects of maize straw extract on average seedling height and biomass under various salt stress conditions. .

Salt level	Treatment	Plant height (cm plant^-1^)	Shoot mass (g plant^-1^ DW)	Root mass (g plant^-1^ DW)	Root mass:shoot mass
None	CK	3.24^a^	0.208^a^	0.151^ab^	0.78:1
	STL	2.62^b^	0.202^a^	0.161^a^	0.81:1
	STH	2.75^b^	0.206^a^	0.162^a^	0.79:1
Low	SAL	2.34^c^	0.180^ab^	0.129^bc^	0.74:1
	SAL-STL	2.35^c^	0.168^bc^	0.128^bc^	0.74:1
	SAL-STH	2.18^c^	0.177^ab^	0.125^bc^	0.72:1
High	SAH	2.14^c^	0.130^d^	0.097^c^	0.76:1
	SAH-STL	1.64^d^	0.140^cd^	0.111^c^	0.81:1
	SAH-STH	1.78^d^	0.137^cd^	0.116^c^	0.86:1

Different lowercase letters indicate significant differences.

### 3.3 Root morphology and activity

**Table 5** shows there were no significant differences in any mo rphology measurements among CK, STL, and STH apart from plant volume, which was significantly larger for CK (0.103 cm^3^ plant^-1^) than STL and STH (0.040-0.058 cm^3^ plant^-1^). Those treatments without salt stress had remarkably longer roots (28.2-33.8 cm plant^-1^) than treatments under salt stress conditions (12.9-25.6 cm plant^-1^), all significant differences apart from the difference between STL and SAL-STH. Under low salt stress, SAL-STH had significantly longer roots (25.6 cm plant^-1^) than SAL and SAL-STL (12.9-16.4 cm plant^-1^). This trend was also observed in plant surface area, with SAL-STH being significantly larger (3.27 cm^2^ plant^-1^) than SAL-STL and SAL (1.66-2.13 cm^2^ plant^-1^). Under high salt stress, root morphology measurements further declined compared to CK, particularly in the SAH-STH treatment (13.6 cm plant^-1^). Root surface area and volume also decreased. Nevertheless, there were no significant differences in morphology measurements between SAH and SAH-STL or SAH-STH treatments. Under low salt conditions, the high-concentration straw extract significantly promoted total root length and root surface area. This suggests that it can induce root morphological plasticity to enhance water and nutrient uptake capacity.

**Table 5:** Effects of maize straw extract on seedling root morphology under various salt stress conditions.

Salt level	Treatment	Total root length (cm plant^-1^)	Root surface area (cm^2^ plant^-1^)	Root volume (cm^3^ plant^-1^)	Root average diameter (mm)
None	CK	32.0^a^	3.05^ab^	0.103^a^	0.566^a^
	STL	28.2^ab^	2.3^abc^	0.058^bc^	0.562^ab^
	STH	33.8^a^	3.29^a^	0.040^c^	0.551^ab^
Low	SAL	16.4^cd^	2.13^abc^	0.045^c^	0.561^ab^
	SAL-STL	12.9^d^	1.66^c^	0.040^c^	0.553^ab^
	SAL-STH	25.6^b^	3.27^a^	0.040^c^	0.531^b^
High	SAH	18.2^c^	2.18^abc^	0.059^bc^	0.568^a^
	SAH-STL	18.3^c^	1.85^bc^	0.079^ab^	0.549^ab^
	SAH-STH	13.6^cd^	1.86^bc^	0.066^bc^	0.549^ab^

Different lowercase letters indicate significant differences (*p* < 0.05).

### 3.4 Root activity reduction

**Figure 1:**
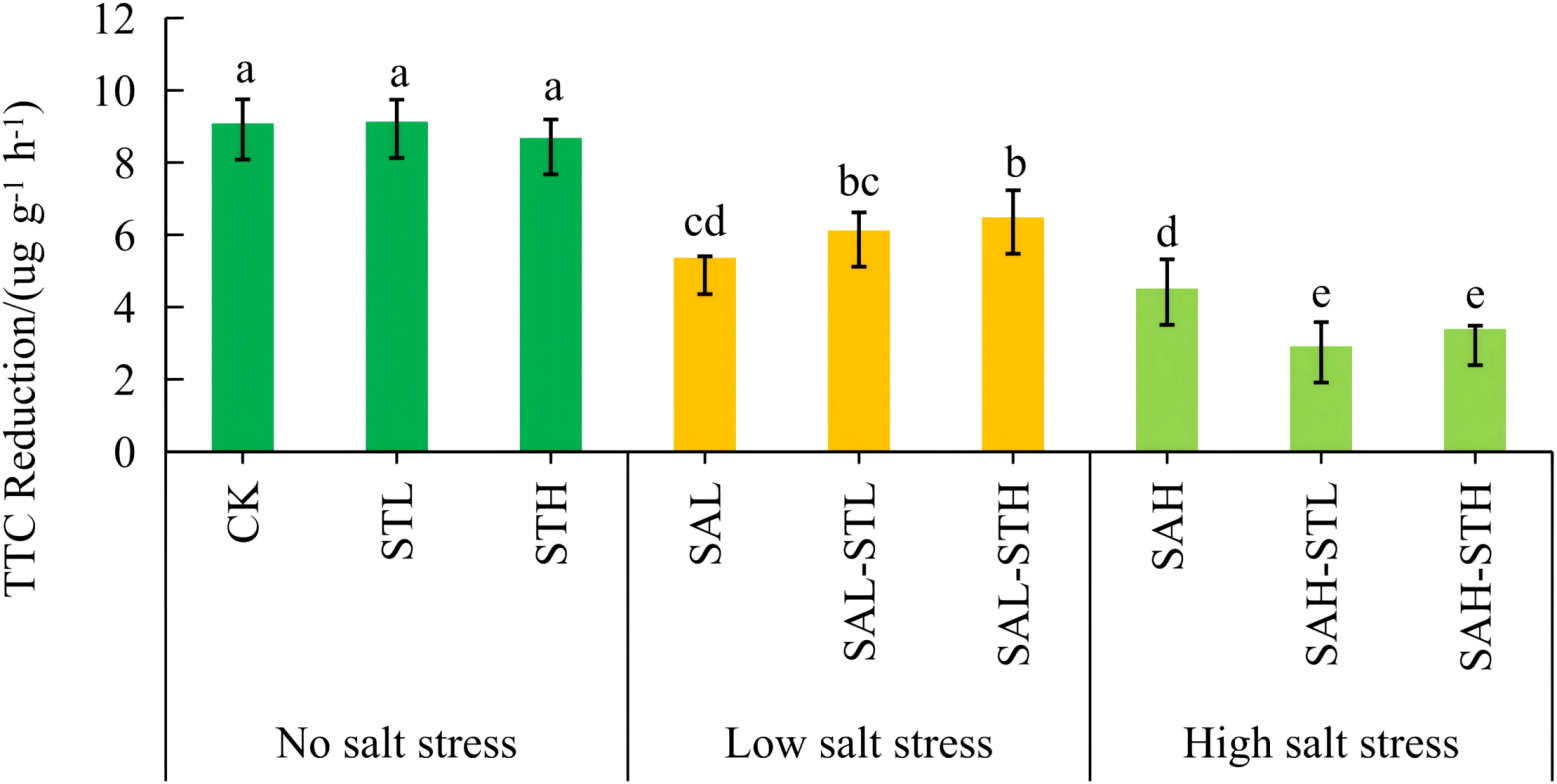
Effect of maize straw extract on seedling 2,3,5-triphenyltetrazolium chloride (TTC) reduction under various levels of salt stress, with n = 3 and error bars representing SD. Different lowercase letters indicate significant differences (*p* < 0.05).

**Figure 1** shows that TTC reduction levels in STL, STH, and CK were not significantly different. However, these treatments had significantly higher TTC reduction levels (8.67-9.13 μg g^-1^ h^-1^) than all treatments under salt stress (2.91-6.47 μg g^-1^ h^-1^). Under low salt stress conditions, the SAL-STH treatment had significantly higher TTC reduction (6.47 μg g^-1^ h^-1^) than SAL (5.36 μg g^-1^ h^-1^). On the other hand, under high salt stress conditions, the TTC reduction observed for SAH-STL and SAH-STH (2.91-3.39 μg g^-1^ h^-1^) was significantly lower than SAH (4.51 μg g^-1^ h^-1^). The increased TTC reduction under the SAL-STH treatment further confirms that root metabolic activity was improved. However, under high salt stress, the straw extract did not exert a similar effect.

### 3.5 Malondialdehyde content

**Figure 2:**
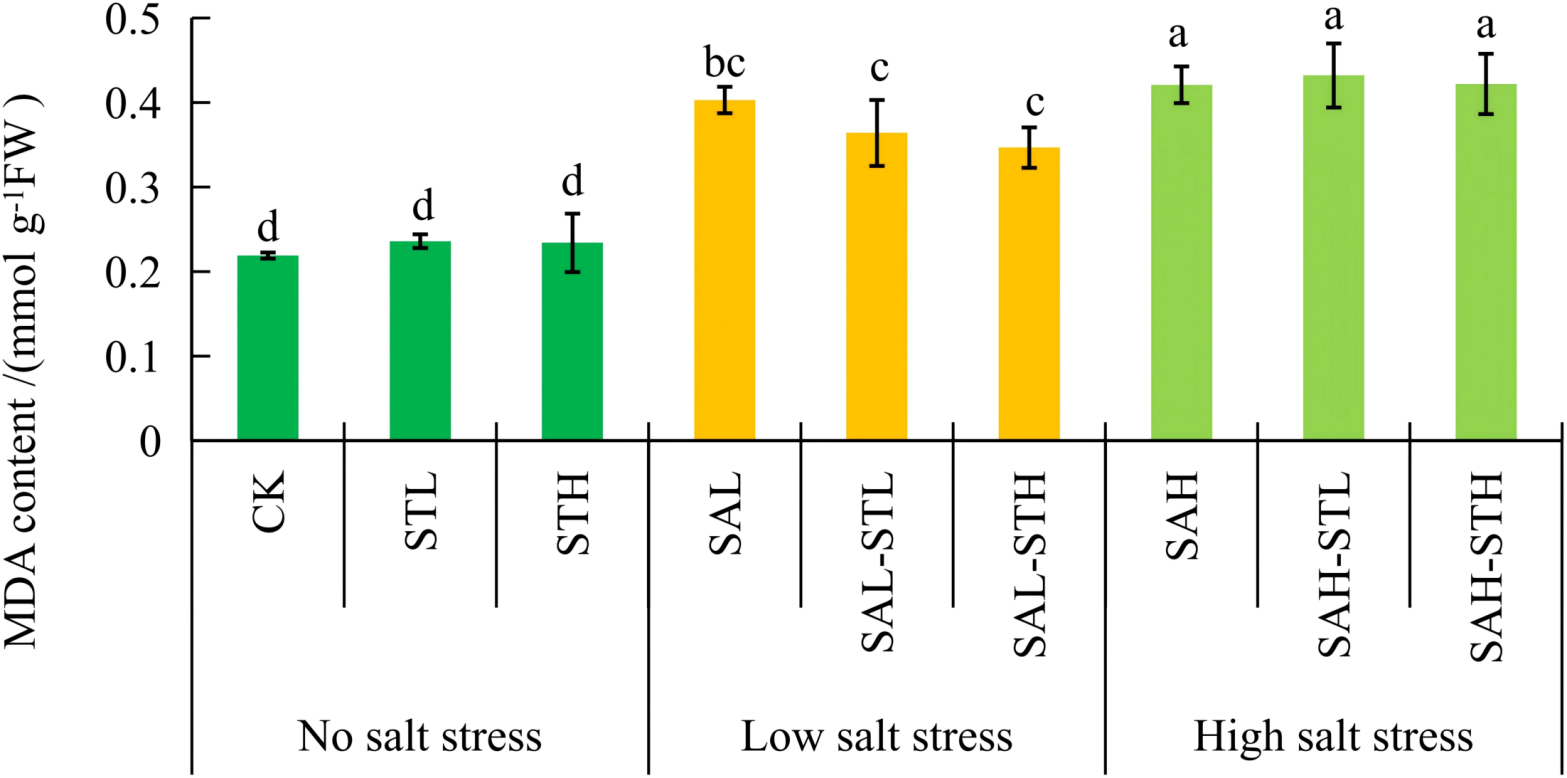
Effect of maize straw extract on seedling malondialdehyde (MDA) content under various levels of salt stress, with n = 3 and error bars representing SD. Different lowercase letters indicate significant differences (*p* < 0.05).

**Figure 2** shows that the MDA content in CK, STL, and STH treatments was significantly lower than in all salt-stressed treatments. Among the low salt-stressed treatments, the MDA content observed in the treatment with high-concentration straw extract was 14.0% lower than in the SAL treatment, a significant difference (*p* < 0.05). No significant differences in MDA content were observed among the three treatments at high salt concentrations (SAH, SAH-STL, and SAH-STH). Under the SAL-STH treatment, the reduction in MDA content indicates that the high-concentration straw extract effectively alleviated lipid peroxidation damage to cell membranes under moderate salinity.

### 3.6 Antioxidant enzymes

**Figure 3:**
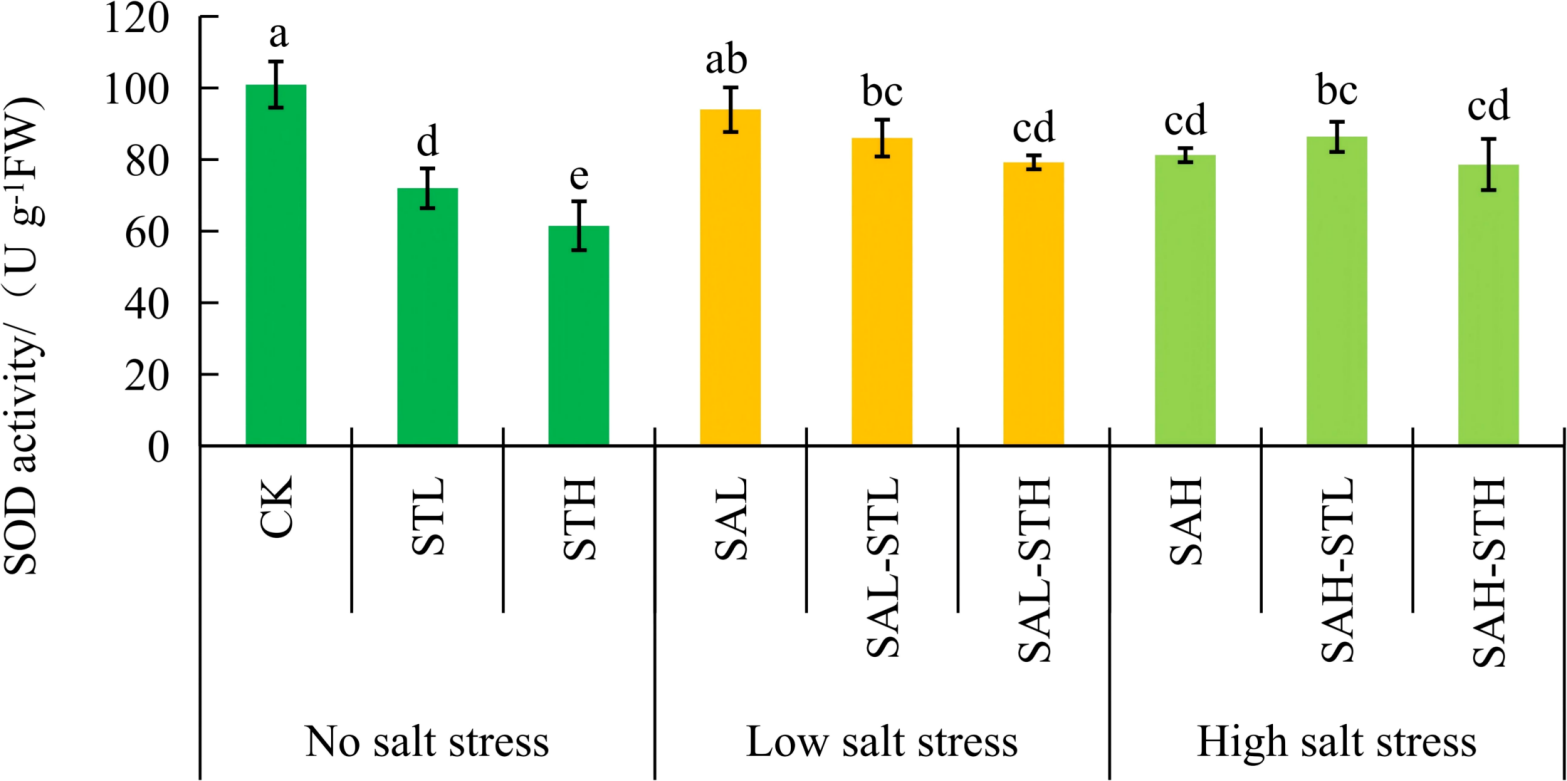
Effects of maize straw extract on seedling Superoxide dismutase (SOD) activity under various salt stress levels, with n = 3 and error bars representing SD. Different lowercase letters indicate significant differences (*p* < 0.05).

**Figure 4:**
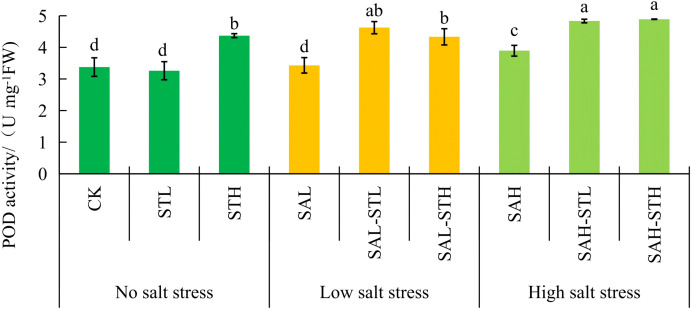
Effects of maize straw extract on seedling peroxidase (POD) activity under various salt stress levels, with n = 3 and error bars representing SD. Different lowercase letters indicate significant differences (*p* < 0.05).

**Figure 3** shows that SOD activity in the CK treatment was significantly higher than the STL and STH treatments. SOD in the CK was not significantly different from SAL, but it was 17.4%, 27.4%, 16.9%, and 28.4% higher than in SAL-STL, SAL-STH, SAH-STL, and SAH-STH, respectively, which are all significant differences (*p* < 0.05). Under low salt stress conditions, a significant (*p < 0.05*) reduction in SOD activity was observed when the higher concentration straw extract was applied (SAL-STH) compared to SAL. No significant difference was observed between SAL-STL and SAL. Under high salt stress conditions, there were no significant differences between SAH and SAH-STL or SAH-STH. Under the SAL-STH treatment, the decrease in SOD activity was consistent with the trend of decreasing MDA content. This indirectly indicates that the level of oxidative stress was alleviated. **Figure 4** shows that POD activity of the CK was not significantly different from STL but was significantly lower than that observed in the STH treatment. POD activity under low salt stress was significantly (*p* < 0.05) lower in the SAL-STH treatment compared to SAL without straw extract. Under high salt stress, POD activity was significantly (*p* < 0.05) higher in both straw extract treatments compared to SAH. Under high salt stress, the straw extract treatments significantly increased POD activity. This may help scavenge excess reactive oxygen species.

### 3.7 Osmotic regulators

#### 3.7.1 Soluble sugar and proline content

**Figure 5:**
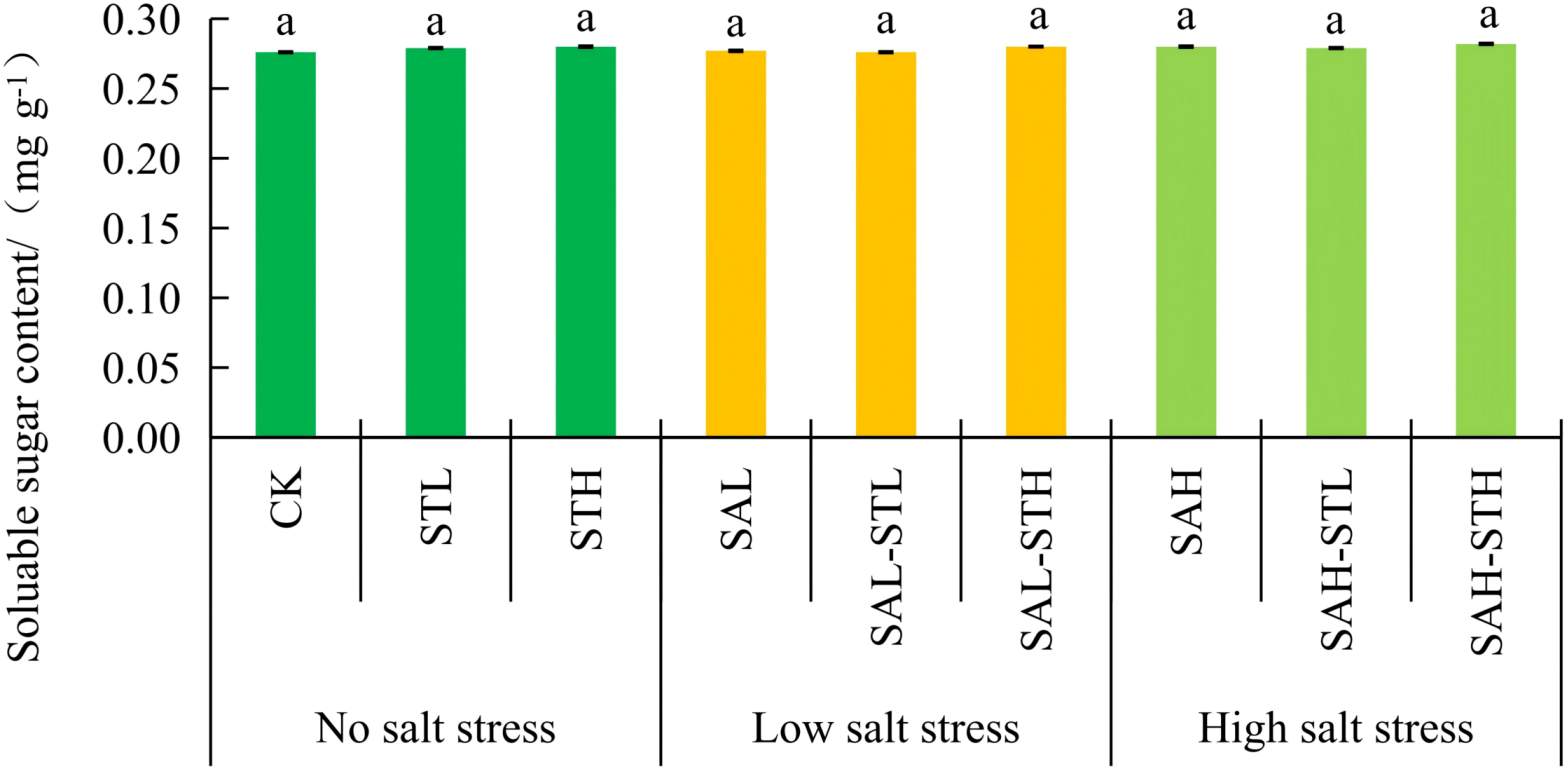
Effects of maize straw extract on seedling soluble sugar content under various salt stress levels, with n = 3 and error bars representing SD. Different lowercase letters indicate significant differences (*p* < 0.05).

**Figure 6:**
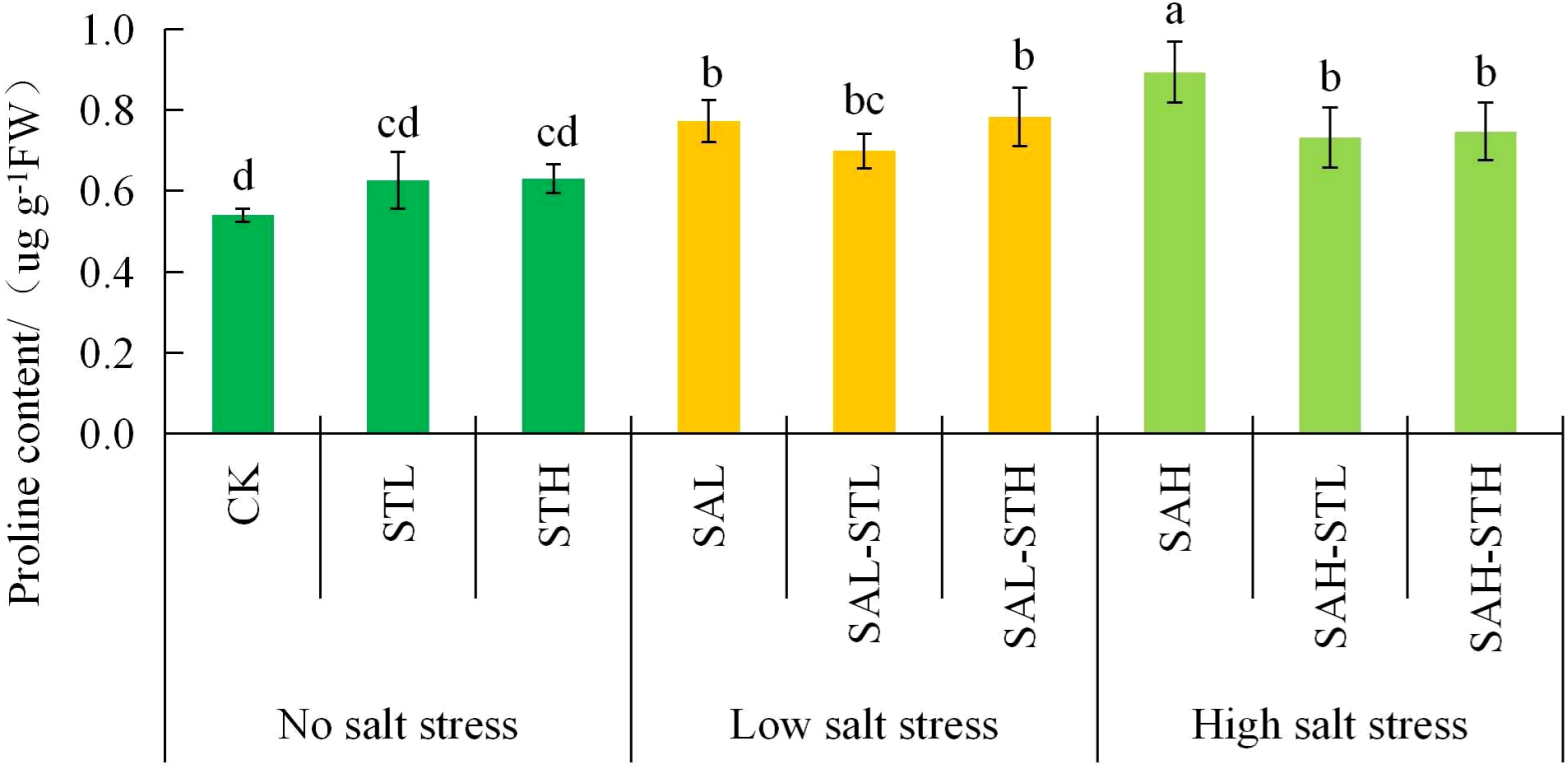
Effects of maize straw extract on proline content of wheat seedlings, with n = 3 and error bars representing SD. Different lowercase letters indicate significant differences (*p* < 0.05).

**Figure 5** shows no significant differences in soluble sugar content among any treatments, which were unaffected by either the salt stress level or the use of straw extract. This indicates that soluble sugar was not the primary osmotic regulator under the conditions of this experiment. **Figure 6** shows no significant difference (*p*>0.05) in proline content between the CK and seedlings treated with maize straw extract without salt stress. However, the proline content of seedlings under salt stress (SAL, SAH) was significantly higher than that of the CK. Under low salt stress conditions, no significant differences were found between SAL, SAL-STL or SAL-STH. At high salt stress conditions, both straw extract treatments (SAH-STL and SAH-STH) had significantly lower proline content than SAH (18.0%, and 16.3% lower, respectively). Under high salt stress, the straw extract treatments significantly reduced proline content. This indicates that the osmotic stress experienced by the seedlings was alleviated. This finding is consistent with the improvement in ion balance.

#### 3.7.2 Ion balance

**Table 6** shows that salt stress led to significantly increased Na^+^ content in all treatments, with Na^+^ in the low salt stress seedlings in the range 22.2-25.38 mg g-1 and high salt stress seedlings in the range 28.35-32.05 mg g-1 compared to 1.95 mg g-1 for CK. Under no salt stress, the K^+^ content in both low and high straw extract treatments (68.29 and 66.75 mg g⁻¹ DW, respectively) significantly increased compared to the control (54.79 mg g⁻¹ DW). Similar trends were observed under low and high salt stress conditions, where straw extract treatments led to higher K^+^ content than seedlings without straw extract, with high extract consistently showing the most improvement. The highest K^+^ level was observed with high straw extract under no salt stress, and the lowest was in seedlings without straw extract under high salt stress. The Ca^2+^ and Mg^2+^ content under salt stress was significantly lower than that of CK, STL, and STH treatments, with levels decreasing notably with increasing salt concentrations. The Mg^2+^ content in SAH-STH treatment was significantly higher than SAH-STL treatment.

**Table 6:** Effects of maize straw extract on the ion balance of seedlings under various salt stress levels.

Salt level	Treatment	Na+ (mg g^-1^ DW)	K+ (mg g^-1^ DW)	Ca2+ (mg g^-1^ DW)	Mg2+ (mg g^-1^ DW)	K+/Na+	Ca2+/Na+	Mg2+/Na+
None	CK	1.95^e^	54.79^b^	8.85^a^	7.19^a^	27.00^c^	4.53^b^	3.70^b^
	STL	1.83^e^	66.75^a^	8.73^a^	6.87^a^	36.54^b^	4.78^b^	3.76^b^
	STH	1.85^e^	68.29^a^	8.57^a^	7.48^a^	44.46^a^	5.59^a^	4.86^a^
Low	SAL	22.21^d^	36.45^d^	6.45^b^	4.44^b^	1.64^f^	0.29^cd^	0.20^c^
	SAL-STL	25.38^c^	52.14^b^	6.58^b^	5.10^b^	2.06^e^	0.26^d^	0.20^c^
	SAL-STH	22.41^d^	56.30^b^	6.75^b^	4.73^b^	2.52^d^	0.30^c^	0.21^c^
High	SAH	32.05^a^	28.57^e^	5.73^c^	2.52^d^	0.89^h^	0.18^e^	0.08^e^
	SAH-STL	30.40^ab^	36.76^d^	5.25^c^	2.65^d^	1.21^g^	0.17^e^	0.09^e^
	SAH-STH	28.35^b^	42.79^c^	5.46^c^	3.48^c^	1.52^f^	0.19^e^	0.13^d^

Single factor analysis of variance (ANOVA) was performed after logarithmic transformation. Different lowercase letters indicate significant differences (*p* < 0.05).

Based on the above, salt stress significantly reduced ion ratios (K^+^/Na^+^, Ca^2+^/Na^+^, Mg^2+^/Na^+^), whereas K^+^/Na^+^, Ca^2+^/Na^+^, and Mg^2+^/Na^+^ ratios in STH treatment were significantly higher than other treatments, with STL treatment also higher. K^+^/Na^+^ and Ca^2+^/Na^+^ ratios in SAL-STH were significantly higher than SAL-STL, while K^+^/Na^+^ and Mg^2+^/Na^+^ ratios in SAH-STH were significantly higher than SAH-STL. Straw extract treatments significantly increased the K^+^/Na^+^, Ca^2+^/Na^+^, and Mg^2+^/Na^+^ ratios. This indicates that they help maintain ion homeostasis. This may be one of the key mechanisms by which straw extract alleviates salt stress.

### 3.8 Correlation analysis

**Figure 7:**
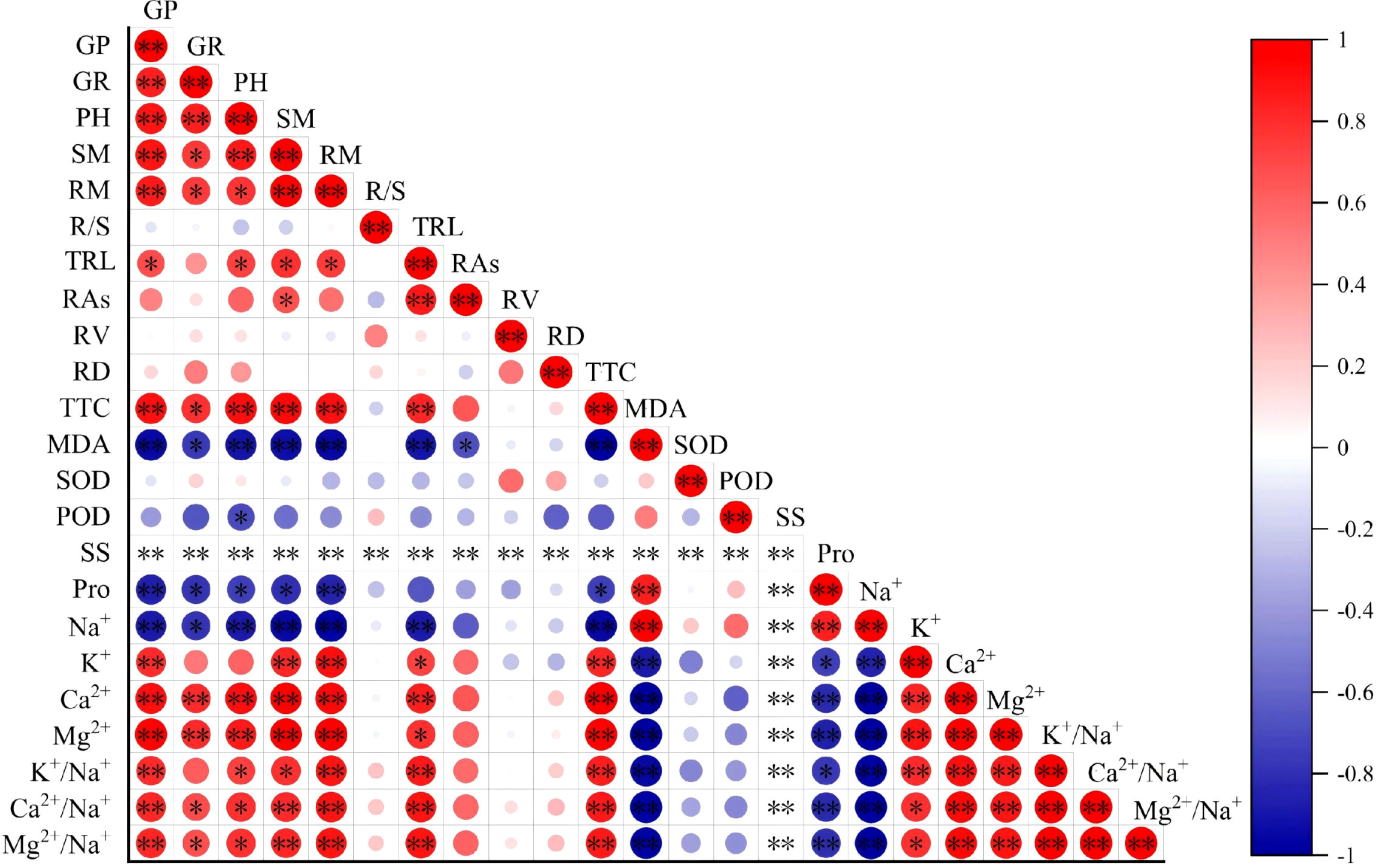
Correlation analysis of growth and physiological indices of wheat seedlings under maize straw extract treatment and salt stress. GP, Germination potential; GR, Germination rate; PH, Plant height; SM, Shoot dry weight; RM, Root dry weight; R/S, Root-to-shoot ratio; TRL, Total root length; RAs, Root surface area; RV, Root volume; RD, Root average diameter; TTC, Root activity; MDA, Malondialdehyde content; SOD, SOD enzyme activity; POD, POD enzyme activity; SS, Soluble sugar content; Pro, Proline content; Na^+^, Sodium ion content; K^+^, Potassium ion content; Ca^2+^, Calcium ion content; Mg^2+^, Magnesium ion content; K^+^/Na^+^, Potassium-to-sodium ion ratio; Ca^2+^/Na^+^, Calcium-to-sodium ion ratio; Mg^2+^/Na^+^, Magnesium-to-sodium ion ratio. * indicates significant correlation at *P* ≤ 0.05 level, ** indicates significant correlation at *P* ≤ 0.01 level.

Germination potential, germination rate, plant height, seedling dry weight, and root dry weight exhibited significant negative correlations with Na^+^ content, indicating that salt stress significantly inhibited wheat growth (**Figure 7**). In contrast, MDA content showed a significant positive correlation with Na^+^ content, suggesting that salt stress increased the degree of lipid peroxidation in cell membranes, leading to cellular damage. Proline content was significantly positively correlated with Na^+^ content but negatively correlated with morphological indicators and TTC, indicating that proline likely serves as one of the osmoregulatory substances in wheat seedlings under salt stress. Growth indicators were significantly positively correlated with K^+^, Ca^2+^, Mg^+^, K^+^/Na^+^, Ca^2+^/Na^+^, and Mg^2+^/Na^+^ ratios. Similarly, straw extract treatments (STL, STH, SAL-STL, SAL-STH, SAH-STL, SAH-STH) showed significant positive correlations with Ca^2+^/Na^+^, and Mg^2+^/Na^+^ ratios, demonstrating that straw extracts can improve ion homeostasis in wheat, enhance salt tolerance, and promote growth. The correlation analysis further confirms that maintaining a higher K^+^/Na^+^ ratio is closely associated with improved growth indicators. In addition, the positive effects of straw extract are highly correlated with its ability to improve ion balance.

## 4 Discussion

The germination potential and rate are primary indicators of seedling establishment, which are negatively impacted by salt stress conditions (Dong et al., 2019; Jovičić et al., 2025; Zhou et al., 2025). Our results show that neither the low nor high-concentration straw extract significantly improved germination rates or germination potential in either low or high salt stress conditions. In fact, the SAL-STH treatment decreased the germination rate significantly lower than SAL-STL but not significantly different from SAL. It is unclear why the germination rate dropped in this particular case. Alam et al. (2002), also reported that lambsquarters (*Chenopodium album* L.; an amaranth family member) extracts had no alleviation effect on wheat germination under salt stress. Furthermore, a high-concentration rice straw extract (60 g L^-1^) was even found to inhibit wheat seed germination (Li et al., 2024). On the other hand, Khoso et al. (2017) and Elrys et al. (2020) did find a mitigation effect using weed extract.

The wheat straw extract also had no significant effect on plant height or biomass. Under high salt stress, the SAH-STL and SAH-STH treatments had smaller average heights than the plant height in SAH, showing the straw extract had a negative effect on plant height. Shoot and root mass were lower in the SAH treatment compared to SAH-STL and SAH-STH, although the differences were not significant. Hamideh et al. (2015) showed that different concentrations of redroot pigweed extract significantly reduced the shoot length and root length and increased the root-shoot ratio of wheat but had no significant effect on the total dry matter and relative growth rate. It has also been reported that higher concentrations (10% and 15%) of *Galinsoga parviflora* (Cav.) and *Oxalis fontana* aqueous extracts increased the elongation and dry weight of *Raphanus sativus* (L.) seedlings (Katarzyna et al., 2018).

Generally, crop roots demonstrate adaptive growth, and their morphological indexes directly indicate crop growth potential (Rawat et al., 2017). In this study, under low salt stress conditions, the application of higher concentration straw extract resulted in notably longer roots compared to the SAL and SAL-STL treatments. This pattern was also evident in terms of plant surface area. However, under conditions of high salt stress, no significant disparities in morphological measurements were observed between the SAH treatment and the SAH-STL or SAH-STH treatments. Aslam et al. (2017) showed that low extract concentrations promoted root growth of alfalfa (*Medicago sativa* L.). This result is also consistent with Chen et al. (2016), who studied the effect of maize straw extract on root system characteristics of wheat seeding. Significant differences in root morphology, particularly in root length, were observed between SAL and SAL-STH treatments, consistent with the findings of Wang et al. (2014) on *Sphaerophysa salsula* (Pall.). Wang et al. (2019) showed that the straw extract of pea (*Pisum sativum* L.) had little effect on shallow roots but significantly increased the biomass and length density of deep roots.

We further consider that this pattern—reduced plant height but enhanced root growth under low salt conditions—may reflect a resource allocation strategy in wheat under salt stress. This strategy prioritizes root investment to enhance water and nutrient uptake, even at the expense of aboveground growth. This trade-off mechanism has been widely documented in salt stress studies (Roussos, 2023). Regarding whether this effect is allelopathic or nutritional in nature, we propose that allelopathic effects likely play a more dominant role. First, indole derivatives (e.g., indole-3-acetic acid and indole-3-butyric acid) present in the straw extract have been shown to promote root growth (Butova et al., 2023). Second, if a nutritional effect were primarily responsible, enhanced root growth would likely be accompanied by improved aboveground growth, which was not observed in this study. While we cannot completely exclude a synergistic contribution from micronutrients in the extract, their role appears limited.

Malondialdehyde content reflects plant cell membrane damage, antioxidant capacity, and the plant’s stress level (He et al., 2025; Sah et al., 2025). In this study, under low salt conditions, the treatment with high-concentration straw extract brought about a 14.0% lower MDA content than the SAL treatment.The increased POD activity may contribute to the lower MDA content (**Figure 4**). In addition, guaiacol identified in the straw extract is a natural antioxidant. It is catalyzed by POD to eliminate H_2_O_2_, which may help protect plasma membrane stability and reduce MDA production (Kesawat et al., 2023). However, no significant differences in MDA content was observed among treatments at high salt stress conditions. This may be because high salt stress exceeded the regulatory capacity of both wheat seedlings and the straw extract.

Additionally, crops respond to salt stress by increasing the activities of antioxidant enzymes and the content of osmotic regulators to alleviate cell membrane damage (Zhang et al., 2026). In this study, the POD activity of wheat treated with straw under salt stress (SAL-STL, SAL-STH, SAH-STL, and SAH-STH) was significantly higher than CK. Considering the differences in MDA content among the treatments, POD may be one of the antioxidant enzymes involved in wheat seedlings’ response to salt stress. Furthermore, a higher concentration of maize straw extract induced POD activity under the STH, SAL-STH, and SAH-STH treatments. This induction may be linked to specific semi-volatile organic compounds detected in the straw extract. These include methyl cinnamate, ethyl cinnamate, and indole derivatives such as indole-3-acetic acid, all of which have been reported to activate antioxidant enzyme activities (Zuo et al., 2022; Chen et al., 2024). Abenavoli et al. (2006) reported on durum wheat where allelochemicals released from sugarcane straw induced proline accumulation, but, maize straw extract did not increase the proline content in wheat during the seedling stage in this study. However, Sadeghi and Bazdar (2018) reported that different concentrations of Bupleurum extracts increased the proline content of *T. resupinatum* (L.). We observed that salt stress caused the accumulation of proline. In contrast, the proline content in straw extract-treated seedlings under salt stress was lower than that under high salt stress alone. This may be because maize straw extract alleviated salt stress through enhanced POD activity (**Figure 4**) and maintenance of ion homeostasis (**Table 6**). Consequently, the stress level may not have reached the threshold for proline accumulation. Alternatively, the active components in the straw extract may have inhibited proline synthesis and accumulation. Feng et al. (2026) observed a similar pattern when treating dandelion (*Taraxacum mongolicum* Hand.-Mazz.) with Pagoda Tree (*Sophora japonica* L.) leaf litter extract. At high salt stress conditions, both straw extract treatments (SAH-STL and SAH-STH) had significantly lower proline content than SAH (18.0% and 16.3% lower, respectively), showing a significant alleviation effect from the straw extract.

Under salt stress, the concentration of Na ions in plants increases significantly, while the absorption of beneficial cations such as potassium, calcium, and magnesium are inhibited. This imbalance disrupts ion homeostasis, negatively impacting physiological metabolism and hindering plant growth and development (Cebeci et al., 2025). In our study, Na^+^ content increased significantly under salt stress, K^+^ content increased with straw extract treatments under both salt stress and no salt stress conditions. Both Ca^2+^ and Mg^2+^ content decreased notably under salt stress. Mg^2+^ content was higher in the SAH-STH treatment compared to the SAH-STL treatment. This suggests that the addition of straw extract promoted the absorption of potassium ions by wheat seedlings. This is likely due to the high potassium content in the straw extract. Additionally, compounds such as ethyl cinnamate and methyl cinnamate identified in the extract may upregulate the expression of vacuolar Na^+^/H^+^ antiporters *SOS1* and *NHX1*. This plays a critical role in maintaining cellular ion homeostasis (Chen et al., 2024), especially at the higher concentration (Hasnain et al., 2023). A higher K^+^/Na^+^ ratio helps maintain ionic balance, reducing magnesium ion precipitation, as indicated by the higher magnesium ion content and Mg^2+^/Na^+^ ratio in the SAH-STH treatment compared to SAH-STL (Abdel Latef et al., 2021). The ability of straw leachate to maintain elevated K^+^/Na^+^ and Mg^2+^/Na^+^ ratios in wheat seedlings may represent a potential advantage of maize straw incorporation for the remediation of saline-alkali soils.

## 5 Conclusion

In conclusion, while both low and high-concentration straw extracts did not bring about significant improvements in germination potential or rate, they did demonstrate a mitigation effect on root morphology and antioxidant enzyme activity, especially under low salt stress conditions. The high-concentration straw extract notably enhanced total root length and facilitated K^+^ absorption, potentially contributing to enhanced stress tolerance. Nonetheless, it also resulted in adverse effects on plant height and displayed inconsistent effects on parameters such as MDA and proline content. Promisingly, at high salt stress conditions, straw extract treatments brought about significantly lower proline content showing a significant alleviation effect from the straw extract. However, this study was conducted under hydroponic conditions over a relatively short period (10 days). To fully evaluate the overall effects of the treatments, further research in saline-alkali soil over a longer growth period is necessary. Soil conditions can alter the relationships among different parameters and provide a more realistic assessment of the practical application of maize straw extract.

## Data Availability

The original contributions presented in the study are included in the article/supplementary material. Further inquiries can be directed to the corresponding author.
